# Beyond the surface: A case study about nevus sebaceous removal for cosmetic concerns

**DOI:** 10.1177/2050313X251317802

**Published:** 2025-02-10

**Authors:** Joshua Lewis, Jatinder P Singh, Ernst J Nicanord

**Affiliations:** 1 John Sealy School of Medicine, University of Texas Medical Branch, Galveston, TX, USA; 2 Department of Family Medicine, University of Texas Medical Branch John Sealy Hospital, Galveston, TX, USA

**Keywords:** Nevus sebaceous, cosmetic, biopsy, birthmark

## Abstract

Nevus sebaceous, a benign scalp birthmark, typically presents without immediate health concerns but may require removal as it becomes bothersome with age. Symptoms are typically cosmetic, lacking pain or bleeding. Clinical presentation varies with age, initially appearing as a smooth, hairless yellowish patch, later evolving into an elevated lesion after the onset of puberty. We report a case of a 25-year-old female with nevus sebaceous. Despite its benign nature, the patient opted for excision due to cosmetic concerns and the potential for malignancy. Surgical removal was successful without complications. This case underscores the importance of early diagnosis and intervention to alleviate discomfort and potential malignancy. While rare, malignant transformation can occur, emphasizing the need for vigilant monitoring and timely surgical intervention, particularly during pre-puberty stages. Treatment should prioritize removal to prevent malignant progression and address cosmetic concerns. Further research is needed to refine management strategies for nevus sebaceous.

## Introduction

Nevus sebaceous is a birthmark usually located on the scalp.^1^ A nevus sebaceous usually does not have any negative adverse effects; however, as one ages, a nevus sebaceous may have to be removed due to the skin lesion becoming bothersome.^2^ The symptoms associated with a nevus sebaceous are cosmetic, lacking pain or bleeding. Nevus sebaceous is usually present at birth and involves many parts of the skin such as the epidermis, sebaceous glands, hair follicles, apocrine glands, and connective tissues.^2^ The presentation of a nevus sebaceous is different depending on the patient’s age. At a young age, nevus sebaceous can appear as a yellowish patch of skin that is smooth and hairless. However, once puberty starts, nevus sebaceous presents as an elevated skin lesion that will grow in size as one matures.^3^ The authors present a case of nevus sebaceous to highlight the clinical findings.

## Case

**Figure 1.**
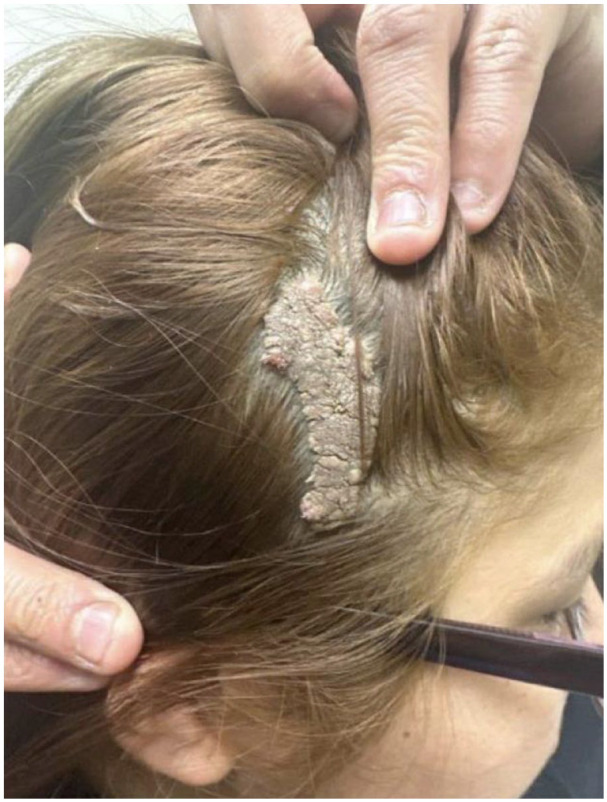
Initial presentation of skin lesion. Brown elevated verrucous growth to the right parietal scalp. No protruding hairs. Not ulcerated or bleeding at this time.

A 25-year-old Caucasian, Hispanic/Latino female presented to the clinic in excellent health with a lesion on her scalp. The growth on her scalp presented as a brown elevated verrucous growth to the right parietal scalp. She had no ulcerated, bleeding, or protruding hair at the time of presentation (Figure 1). She was born with this birthmark, which was initially presented as a “red flat mark” across her scalp. Previously, the birthmark was not bothersome, evaluated, or diagnosed. After graduating high school, she describes how the growth on her scalp has started to grow and has become bothersome during daily activities such as brushing her hair. However, the lesion on her scalp does not bleed or hurt when unbothered. At her initial visit, she was diagnosed with nevus sebaceous because of presentation and childhood history. However, dermatology was constructed to ensure the lesion was malignant. Dermatology confirmed the diagnosis of nevus sebaceous with histopathology and referred the patient to the plastic surgery department.

The Plastic Surgery Department discussed with the patient that the lesion is currently benign, as well as that it has a low malignant potential. However, they reassured the patient that the lesion would not resolve on its own. In addition, the patient desired to have the nevus sebaceous removed because of the “way it looks.” After explaining the risks and the procedure, the surgeon and the patient agreed to have the excision lesion scalp surgery on March 23rd. It was arranged to have the surgical procedure under general anesthesia due to the size of the lesion. She complied with antibiotic coverage and no complications arose.

**Figure 2.**
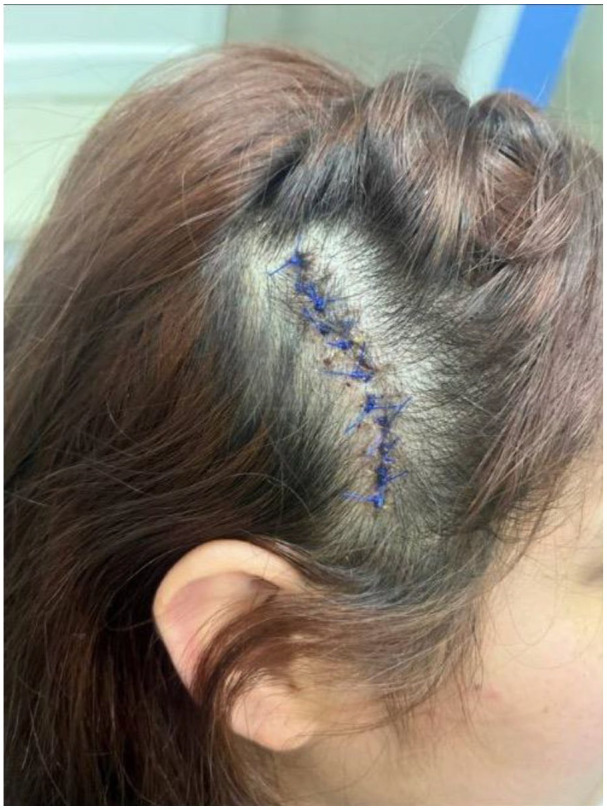
Postoperative photo.

The excision lesion scalp surgery was performed without any complications. The patient’s surgical site did not have any bleeding or hematomas. In addition, her vitals were within normal range post-surgery (Figure 2). At the 3-week follow-up with plastics, the sutures were removed. Bacitracin ointment was applied. In addition, there were no issues after the excision of nevus sebaceous in the scalp.

## Conclusion

We presented a clinical case study of nevus sebaceous. According to the patient’s account, during childhood, there were no discomfort or diagnoses related to the birthmark. However, as she entered puberty and matured, the discomfort associated with the skin lesion intensified and became bothersome. This case underscores the importance for clinicians to consider early diagnosis and treatment of nevus sebaceous, potentially averting adverse effects or discomfort later in life.

## Discussion

Nevus sebaceous commonly affects 0.3% of births, with a presentation as a birthmark.^2^ A nevus sebaceous is commonly classified as a benign tumor; however, it can rarely result in malignancy. Due to this potential concern, it is advised to have it surgically removed upon diagnosis.^4^ In the case of our patient, the birthmark had not been previously evaluated or bothersome. As a result, the lesion could grow in size. The presentation of nevus sebaceous differs based on one’s age. The initial presentations of nevus sebaceous ranges from birth until pre-puberty, with a flat lesion with yellowish-orangish coloration. During this stage, you will not see increased growth of the lesion. However, the onset of puberty will cause the lesion to start to grow in size and thickness, and start having symptoms of itchiness and irritation.^4^

Rare cases of nevus sebaceous have developed into malignant tumors.^5–7^ Malignant tumors have been commonly presented in adult patients, with an increase in incidence with age. However, malignant tumors have not been observed in patients pre-puberty age.^8^ Despite the molecular mechanism of the development of carcinogenesis in the nevus sebaceous, more clinical presentations and initiatives need to be put in place to diagnose this condition at a younger age. Due to

Regarding treatment, excision removal surgery is typically recommended during the pre-puberty stage due to the potential for malignant transformation and the fact that the nevus is not fully developed.^9^ In the case of our patient, despite her age being post-puberty, she did not present with malignant transformation. However, her best option was excision removal surgery to avoid the potential ability of transformation.
